# What do you need to learn in paediatric psycho-oncology?

**DOI:** 10.3332/ecancer.2019.916

**Published:** 2019-03-28

**Authors:** Soumitra Shankar Datta, Tania Saha, Aparupa Ojha, Anirban Das, Rhea Daruvala, Kesavapillai Sukumaran Reghu, Rimpa Achari

**Affiliations:** 1 Department of Palliative Care and Psycho-oncology, Tata Medical Centre, Kolkata 700160, India; 2 EGA UCL Institute for Women’s Health, University College London, London WC1E 6BT, UK; 3 Department of Paediatric Oncology, Tata Medical Centre, Kolkata 700160,, India; 4 Department of Paediatric Haematology, Oncology and Bone Marrow Transplant, Mazumdar Shaw Cancer Centre, Narayana Health City, Bangalore 560099, India; 5 Department of Radiation Oncology, Tata Medical Centre, Kolkata 700160, India

**Keywords:** psycho-oncology, paediatric, children, cancer, anxiety

## Abstract

Paediatric psycho-oncology is an evolving speciality and is increasingly being recognised as an essential component in children’s cancer care. Modern paediatric oncology services aspire to integrate physical care with psycho-social care and build capacity within clinical teams to address the emotional needs of parents and children side by side with other aspects of medical care. This article discusses the unique challenges of paediatric psycho-oncology and common situations where psychological assessment and management of children and young people with cancer become especially important. The authors propose a tiered structure of training. Providing empathic evidence-based psycho-social care is ‘everyone’s business’ in paediatric oncology and not merely that of mental health professionals. However, there are times when a more specialist intervention by a paediatric liaison psychiatrist and/or a clinical psychologist is needed for optimum outcome. Learning interviewing techniques suitable for children and adolescents should be a core part of the training in paediatric psycho-oncology. Professionals should be encouraged to reflect on their own emotional wellbeing, which in turn will provide a stable foundation of emotionally matured care to children, young people and their families.

## Why is training needed in paediatric psycho-oncology?

With the improved survival rates of childhood cancer, it is now considered important to look beyond disease remission [1, 2]. This includes provision of long-term psychological and social care for childhood cancer survivors. There have been consensus statements from professional bodies, highlighting the need to streamline and standardise psychosocial care for children and young people who are cancer survivors [3]. Moving beyond mind-body dualism, the authors of the current article propose that paediatric cancer centres develop a model to deliver collaborative clinical care through joint working between paediatric oncologists and child mental health professionals. Many of the professionals who treat children with cancer have excellent training in dealing with the disease from a medical point of view but not necessarily from a socio-psychological point of view. There are very few training opportunities or initiatives in paediatric psycho-oncology that addresses the training needs of oncology professionals [4]. This article will try to provide a framework for training in paediatric psycho-oncology that will be relevant to most clinicians looking after children with cancer. The readiness to learn about psychological care and adopting the various treatment strategies that is individualised to a particular child and his/her family will likely reap the maximum benefit for the patient.

## Unique psychological challenges in paediatric psycho-oncology

### Pitching communication at the right developmental level

Adult ways of communication are not suitable for children because children perceive the world around them differently [5]. Moreover, the developmental age of the child may be different from his/her chronological age. Children who have had an experience of sickness often have a better idea of health and disease than other healthy children of the same age.

### Dealing with anger and resentment

Adolescence is a period of transition that is characterised by young people experimenting and exploring the world around them, often against the norms set by the adults around them. Teenagers who are medically unwell may resent authority figures who are in control of their lives.

### Dealing with conflicting viewpoints

Parental views may be different from a teenaged person [6]. Clinicians often have to address both these views while treating a young person with cancer. It is good practice to engage the young person in treatment-related decisions. In situations where there are disparate views between the young person and their family members, it is often useful to understand the reason underlying the disagreements. It may be tempting to side with the adult caregivers, but this may result in the young person feeling isolated and cornered. The final outcome of conflict resolution should aim at a win-win situation for all those involved in the conflict.

### Dealing with unconscious identification and blurring of boundaries

Many of the children treated by paediatric oncologists have illnesses that require long-term engagement of the patient with the services, often over several years. This may come with the advantage of knowing a particular child very well, but at the same time having the disadvantage of unconsciously evoking certain unwanted negative or positive emotions within the clinician’s mind. Children may also view the clinical specialist as a member of his/her extended family and this may blur boundaries and hamper objective assessments.

### Caring for a child who is dying

Sometimes even scientifically well-informed medical networks are uncomfortable in accepting that some children diagnosed with a life-limiting illness may die. Caring for a dying child is emotionally challenging. It is important to offer good quality palliative care to these children and young people. It is now acknowledged that paediatric palliative care physicians need to be extremely emotionally skilled [7]. The other challenges of paediatric palliative care are to do with managing strong emotions that may emerge within the families and staff groups, having adequate expertise to handle specific symptoms such as pain in children and integrating physical and psychological aspects of care. The views expressed by the young person and their parents should be taken into account in a paediatric palliative care set-up [8].

## Content of a paediatric psycho-oncology training programme

**Figure 1.**
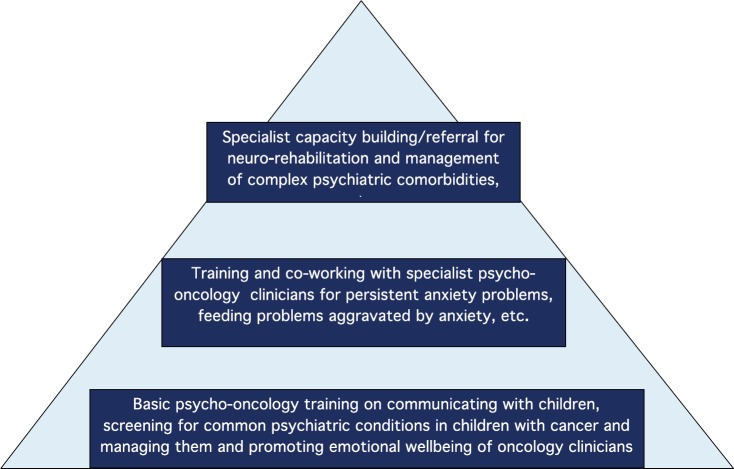
Pyramid of training in paediatric psycho-oncology.

Clinicians who have worked with children with cancer know that broadly there are two groups of scenarios—one when treatment goes according to plan and another when it does not. More skills are needed to handle the second situation than the first. It is important to be nice to children when assessing them, but this may not be sufficient to engage and address all the psychological issues in children with cancer. In fact, too much or too little emotion can hamper the communication process and build mistrust in the doctor–patient relationship. Communicating with children using language appropriate to their developmental level is also important [6]. Recognising the need for psycho-oncology training, we propose that training in paediatric psycho-oncology (Figure 1) may broadly be carried out in three levels:

a) Basic paediatric psycho-oncology training modules: All professionals who work with children with cancer need to have some training in the emotional and behavioural aspects of cancer care. As suggested for training in adult oncology-related communication skills, universal training may be delivered over 3 days [9] for paediatric oncology clinicians until there is a robust evidence base on the format of training that works best. Some centres may choose to incorporate this training as part of their regular appraisal process. The seriousness of the medical illness may not always correlate with the emotional needs of the child. As well as the senior clinicians, the nurses and all the junior doctors also need to able to be communicate with children, teenagers and their family members. They should feel comfortable about discussing the disease, within their respective competencies. Nursing staff often get asked questions that come to the minds of family members once the consultant has left the ward. The basic training modules should focus on learning communication skills for engaging children and adolescents, detecting common mental health issues in children with cancer, managing low threshold and transient emotional difficulties and promoting psychological wellbeing of clinicians. Following completion of generic psycho-oncology training, there can be focused training for advanced learners who are willing to devote the majority of their time to paediatric psycho-oncology.

b) Advanced paediatric psycho-oncology training modules: Specialists and consultants need to be more aware of the finer nuances of psycho-oncology. Modules for more advanced learners can include areas such as dealing with resistant anxiety disorders, managing problems with swallowing and eating that are not explained by any persistent medical causes, handling complex problems that require coordinating with larger multidisciplinary teams, dealing with denial and disparate views of parents, dealing with a depressed adolescent patient with suicidal thoughts, managing a child with cancer and a comorbid psychiatric condition, assessing capacity to consent to treatment for adolescents with cancer, address issues related to non-adherence and providing psychological care at end of life.

c) Specialist paediatric psycho-oncologist training: Some children with more serious emotional problems will require specialist referral who may be a child psychiatrist or a psychologist, who reviews a proportion of children in the unit. A children’s cancer centre will benefit from having a robust paediatric psycho-oncology department. The specialist psycho-oncology training programme is best developed for professionals who already hold a registered qualification in mental health and are interested to work in the field of paediatric psycho-oncology for most of their time. Those professionals who aspire to be paediatric psycho-oncologists would benefit from a period of supervised training in patient management after they have completed a formal qualification in mental health as a clinical psychologist, child psychiatrist or a psychiatric nurse. Paediatric psycho-oncologists are required to be hands-on while managing the neediest and the more emotionally vulnerable children. They should have expertise to manage children with multiple psychiatric comorbidities like conduct disorder with substance misuse in a young person who has to co-operate with a complex cancer treatment regimen. They may often have to manage an angry and bereaved family member of a child who is dying and be able to pull together the team to a consensus.

Asking clinicians about their perceived training needs is a good starting point while establishing a paediatric psycho-oncology training programme. Reviewing video-recorded real-life clinical consultations (after appropriate consent) may give unique insights into the consultation style of individual clinicians and capture their communication strengths and training needs. Patient and parent’s feedback is also useful to identify training gaps. Training is best done through role-playing and other interactive training methods. With children, it is almost impossible to have real patients for training, but one can always have trained actors who act as parents of children with cancer during training sessions. Sharing video-recorded interviews highlighting good clinical practice often initiates beginners to model senior clinicians who are more experienced in handling difficult scenarios.

## Initiating rapport and setting up an interview space

The room in which children are to be interviewed should be comfortable and child-friendly. For children younger than 6 years of age, the clinician should be ready to move around in the interview room if needed to engage with the child. It is usual to have a few toys in the interview room but there should not be too many distractions. Oncology centres should not keep soft toys in clinic rooms as they are seldom washed and may serve as a carrier for infections. Colouring crayons, pen and paper for children to draw and scribble during the interview can be used [10]. Painting/colouring puts the child at ease and reduces the unconscious defences of the child who is being assessed [11]. Being engaged in a task other than answering questions often leads to better rapport with the examiner. The focus of the first interview should be on getting to know the child and need not be very elaborate. Interviewing can start with asking a few routine questions about family or school. Discussing a couple of neutral topics may help as well. Involving the parents is customary as it gives the clinicians valuable information, as well as makes the family empowered. Views expressed by the child are important and efforts should be directed at addressing any specific need voiced by them. One common pitfall while communicating with children is not allowing enough time during the interview.

While interviewing older adolescents, it is important for clinicians to remember that adolescent patients prefer to think in a more mature way and resent being treated like a child. Having the room set-up for small children may annoy teenagers. Several oncology centres around the world have specialised Young Oncology Units that are led by paediatric oncologists having special interest in working with adolescent patients aged 14–18 years. Adolescent patients need the ‘terms and limits of confidentiality’ that are maintained by clinicians to be explained. Some adolescents can be quite direct and discuss their concerns freely. Others may be resentful towards authoritative adults and refuse to engage easily. At other times, clinicians may have to engage the young person alongside his/her family. One also needs to be comfortable in discussing sensitive topics, for example, sexuality and substance misuse if the patient so desires. Clinicians should not be judgemental of the preferences expressed by teenagers, even if they do not conform to the larger societal value system, as long as they do not pose any risk to anyone [12]. A good clinician will make an adolescent feel the need to take responsibility for his or her own actions and draw the young person into the process of care planning and shared decision-making alongside the professionals and the family. If the clinician does not know the answer to a question, it is better to admit that rather than evade the question. Engaging an adolescent with the oncology services is a process, may have its ups and downs, requires constant attention and is a two-way phenomenon. Some of the common communication challenges and ways to tackle them are highlighted in Table 1.

## Learning to break bad news according to developmental age

Most clinicians break bad news to parents of children with cancer. However, breaking the news of cancer to children and young people requires even more skill. A need for better communication with young people with cancer has been identified as a universal need across cultures [13]. Parents are quite protective about children when getting to know about their disease; when it comes to cancer, more so when things are not going according to plan [14]. Children get to know about their disease from all sorts of sources. They could be well informed or misinformed about the disease from other children in the ward, some of whom may have a different diagnosis. In the absence of clear discussions, they may feel lost when they see another child in the ward falling very sick and wrongly personalise this experience. They may guess that the treatment is not going to plan from the body language of their parents or if many people suddenly begin visiting them. One of the authors of the present article had come across a child who got informed about the speciality of the consultant treating him from a television programme in which the doctor had appeared as a specialist. Teenagers prefer to know about their treatment and be involved in advance care planning that obviously needs sharing of the diagnosis and prognosis [15]. This could be because the ‘fear of the unknown’ is usually more intense than ‘known fears’. Before approaching the task of breaking bad news, clinicians need to think about the content of the information they would like to share with a child. Each child is different and will have slightly different information needs. Lastly, it is also important to remember that young people should be given a choice if they want to know the details of their disease and treatment. Occasionally, there are children who prefer to not know, and this choice should be respected [16].

### Communicating with pre-school children

Children aged between 2 and 5 years may not have a very well-developed concept of time, particularly chronology of events. It is therefore appropriate just to share in simple language what is going to happen imminently rather than discussing a long plan of management with these children [5]. To cite an example, for a child who needs to co-operate for a blood test, one may say that ‘a nurse will soon come and meet with you and your mother and she will get a small amount of blood from your body to know about your sickness so that you can get better’.

### Communicating with primary school children

For a 6–11-year-old child, it is important that the clinician remember that at this age, children are interested in and capable of understanding simple biological facts [5]. They appreciate verbal communication but can be quite concrete in their information processing. So, one option is to discuss the disease in simple terms like, ‘Recently, your body is making too many blood cells of one particular kind. This is like weeds growing in a nicely kept garden. So, we would like to give you some medicines that will help to get rid of the weeds’. There are also specially designed cartoons and comics which are quite useful to break news for this age. Example of a children’s book to help understand leukaemia is ‘What’s up with Richard? Medikidz explain Leukaemia’ brought out with the support of Macmillan Cancer Support, CLIC Sargent and Teenage Cancer Trust, UK [17]. There are similar publications for children with osteosarcoma [18].

### Communicating with teenagers

For adolescents 12 years and beyond, it is important to be straightforward in communication [5]. The full course of treatment should be described. As much as possible, clinicians should try to gain the trust of an adolescent patient and stick to their plans. Adolescents hate to be told that they need a couple of days in hospital and then doctors changing their minds halfway through, saying that they might need another 10 days. A better approach is to inform them at admission that it is difficult for the doctors to be precise about the number of days that he or she will be in hospital, but the clinical team will keep the young person informed about the progress. Common side effects of the medication should also be discussed to avoid surprises. With all young people, active listening is important so that they feel that their concerns have been heard.

## Understanding the psychological reactions and difficulties in accepting uncertainties

Paediatric oncologists should be familiar with the various psychological reactions of children and adolescents to their diagnosis of cancer. This can range from complete denial of cancer, acute stress reaction, various other anxiety states, specific procedural anxiety, adjustment disorder, clinical depression and more serious psychological problems as posttraumatic stress disorder and transient psychosis [19–22]. Following diagnosis, invariably there is some degree of uncertainty in the minds of parents regarding the final outcome of the disease. The clinical team has to address disease-related anxiety and stress in close family members of the child during the course of treatment. At the beginning of the treatment, the questions that oncologists grapple with are often to do with the histopathology and biology of the disease, whereas the parents may be thinking ‘will my child survive?’, ‘when will my child be back in school?’, ‘even if she is cured, will she have a normal life?’ or ‘will he or she ever be able to become a parent?’. Thus, one needs to acknowledge the differences in the way various people around the child may react depending on their role in the life of that child. The practice of medicine being an iterative process, it is not unusual for clinicians to discuss an approximate, rather than an exact, timeline of treatment of a particular child with cancer. This uncertainty can make some families upset. Psychological reactions may be coloured by the past experience of dealing with cancer in a different family member, e.g. cancer-related mortality in the extended family may make them unduly pessimistic. In certain societies, the social stigma of cancer can have a significant impact on the child and the way he relates to his environment [23]. In countries where cancer care is not delivered free of cost by the government, families and teenagers could be preoccupied with additional financial worries.

A proportion of children and young people with cancer referred to psycho-oncology services may qualify for a psychiatric diagnosis that requires treatment [24]. The clinical diagnosis may be different for children and young people with various types of malignancies. In the case of haematological malignancies, especially Acute Lymphoblastic Leukamias (ALL) that often constitutes the bulk of referrals, the duration of treatment is long, and the reason for referral may be due to adjustment disorder and procedural anxieties. On the other hand, for young people with sarcoma, the referral may be due to adolescents having body image issues and clinical depression. Table 2 describes the broad psychiatric diagnostic categories of children with a wide variety of cancers seen by the paediatric psycho-oncology team in the institution of the authors over a period of 20 months.

## Procedural anxiety in children with cancer

Training in paediatric psycho-oncology should help clinicians manage anxiety in children with cancer and their parents. Some authors have already suggested universal screening of children, young people and adolescents with cancer for psychological distress [25]. Treatment of childhood cancer can be intense and usually disrupts the normal routine of the child. It is not unusual to find children getting quite anxious during the diagnostic investigations and also during the course of treatment [26]. The anxiety may manifest with recurrent panic attacks, overt expression of fear, avoidance and arousal on hospital visits, all of these becoming particularly intense just before the next episode of care. Clinicians should observe for signs of autonomic arousal and develop an ear to listen to the worries expressed by the child. It is immensely useful to get information about any pre-existing psychiatric problems or vulnerabilities. The assessment for paediatric psycho-oncology should include feedback about the child from nursing staff and paediatricians looking after the child and interacting with the child and his/her caregivers directly. When in doubt, observing the child in neutral situations with no specific triggers for emotional distress can help clinicians differentiate between situational psychological reactions from more pervasive anxiety disorders. Medications that the child is on and other physical parameters should be reviewed to see if organic causes are contributing to the anxiety symptoms. Being treated with steroids can also contribute to mood symptoms. Treatment with antifungal agents like voriconazole, that are known to cause encephalopathy, may be associated with transient neuropsychiatric symptoms [27]. Children with brain tumours may require specialist assessment for cognitive and psychiatric problems.

The cognitive appraisal of cancer being perceived as ‘a life-threatening illness’ by family members can indirectly affect the child’s coping. Some families, on the other hand, may be distressed but try very hard to hide their emotions from the patient. Parents of children with cancer may qualify for a diagnosis of anxiety or depression [28]. The child in this situation picks up on indirect cues from the body language, touch and nonverbal communication patterns of those around him. Thus, parental anxiety alongside the child’s anxiety can influence the psychological health of the child [29]. Clinicians need to learn to address parental anxiety and ideally this topic should be covered in-depth in the paediatric psycho-oncology training course.

The treatment of anxiety disorders is important in children because it usually impacts the management of cancer and may influence treatment adherence. Anticipatory anxiety can be disabling even before the start of a procedure. When natural desensitisation does not happen over time, specialist referral to a child psychiatrist and psychologist is helpful. Psychologically one may use techniques of distraction and hypnosis when dealing with procedural anxiety as highlighted by a Cochrane review on needle-related procedural pain in children and adolescents [30]. Specialists providing psychological treatments for children with anxiety should be proficient in using some of methods of relaxation often used with children, such as guided imagery, play, eye movement desensitisation reprocessing, etc [31]. There is also a role for medications in intractable anxiety that is persistent and interfering in care delivery or when anxiety is impacting the day-to-day life of the child.

**Procedural anxiety**

A 9-year-old school girl diagnosed with leukemia was scared of needles during her second admission. She was referred to the psychooncology team for anxiety related to treatment procedures.

Following a comprehensive psychological assessment, the child was encouraged to express her worries through drawing about her ‘fears’. Guided imagery technique was used to help her relax. The patient was taught to imagine a relaxing experience and think about a ‘safe place’. She chose her bedroom as her ‘safe place’. She was encouraged to imagine the touch of her duvet, colour of her bedroom, as well as the soothing voice of her mother and her best friend speaking to her. Over a week’s time, she was able to control her anxiety and participate in the treatment process more willingly.

## Learning to work in a multidisciplinary way for children with feeding and eating problems

Anxiety in children with cancer may result in them becoming fearful of eating and at times even to speak [32, 33]. The problems often comes to notice after serial examinations of body weight that show weight loss that is not explained medically and at a time when the clinical team may want to wean the child off the nasogastric tube feeding as the child is otherwise well. This may happen with some children with nasopharyngeal cancer or any other type of cancer where the child has been on prolonged nasogastric feeding. In extreme situations, children may become scared to swallow and keep saliva pooled in their mouth. Systematic assessments by specialist paediatric psycho-oncologists is warranted at this point and the interventions have to be coordinated between the clinical nutritionists, child psychologists, child psychiatrists, oncologists, nursing team and the parents. Attitudes towards food and eating, food shopping, choosing the meal times and leisure activities that go on in parallel while eating as a whole are often very much related to the functioning of the family system. Clinicians should thus enquire about who accompanied the child during meal times prior to the diagnosis of cancer, what are the feeding habits of the family in general and what are the parental expectations around eating. Graded introduction of the child to meal times even if this means just sitting with the family members, followed by a liquid diet that the patient can choose to spit out, followed by gentle swallowing of liquids and then moving on to semi-solid and solid food is usually helpful. The above will require systematic intervention according to a pre-designed schedule introduced jointly by a clinical nutritionist and a paediatric psycho-oncologist. Family members need to be supported and their concerns should be addressed as many parents feel very guilty about their child refusing to eat. The consistency and type of food should be culturally appropriate and is best done in consultation with a paediatric clinical nutritionist. Parental involvement should be encouraged so that they feel part of the team supporting the child.

## Managing depression in adolescents with cancer

Children and adolescents with chronic physical illnesses have increased rates of depression [34]. Adolescents typically have poor frustration tolerance and may find it difficult to engage with services, especially at the transition points of cancer care, e.g. at the onset of treatment, at the time of moving back to school or college on treatment completion, at the time of being diagnosed to have recurrence and on moving from curative to palliative treatment [35]. Various concerns voiced by adolescents may have to do with body image like losing hair in response to chemotherapy, having a scar following an abdominal surgery or living with an amputation. Over time, some young people adapt and adjust to the new reality of their life but some may progress to develop clinical depression [36]. Depression in adolescents may be associated with low or irritable mood and frequent anger outbursts. This may be accompanied by biological symptoms (as fragmented/reduced sleep or increased somnolence, increased or reduced appetite), somatic symptoms (as diurnal variation of mood), bodily symptoms (as aches and pains) and cognitive symptoms (as hopelessness or worthlessness). In clinical depression, many of these symptoms are pervasive and impact the social and occupational functioning of the young person. Treatment of depression in adolescents with cancer may need a combination of psychological treatment with antidepressant medication and should be undertaken by specialists trained in the area. Regular risk assessment is essential to monitor and manage risk of completed suicide [37] by trained professionals. Other risks that need to be monitored include the risk of self-harm, the risk of self-neglect and the risk of dropout from treatment.

**Adolescent boy with depression**

A 15-year-old boy with nasopharyngeal cancer was upset about his father’s suicide, which was precipitated after he lost his job due to frequent absences from work while he brought his son for treatment of cancer. The patient complained of feeling very guilty about his father’s death, feeling low in his mood, having disturbed sleep and difficulties in concentration on his studies even after completion of treatment. He also had fleeting suicidal thoughts. Following assessment by the pediatric psycho-oncology team, the patient and his mother were seen together in family therapy sessions. Frequent risk assessment was carried out and risk was managed under the supervision of a consultant child psychiatrist. Psychological therapy sessions by psychologists focused on grief work to achieve closure regarding the loss of a parental figure. The patient was also diagnosed with comorbid depression and he was started on an antidepressant medication (Fluoxetine). Over a period of 6 months of regular follow-up, his depressive symptoms remitted and the patient was able to get back to school. He was no longer suicidal. He again started going out with his peers and could concentrate on his future career.

## Assessing the neuro-cognitive effects of brain tumours and their treatment in children

Paediatric oncologists who treat children with brain tumours are familiar with the neuro-cognitive sequelae of brain tumours and their treatment. The risk factors of neuro-cognitive problems in these children can be child related, disease related or treatment related [38–42]. Being younger (less than 3 years at diagnosis), having co-occurring neuro-sensory deficits, being diagnosed with primary central nervous system (CNS) tumour or a posterior fossa syndrome, having comorbid CNS infections, having a family history of learning or attentional problems, having been treated with total brain irradiation, high-dose methotrexate or ventricular shunt placement are all associated with increased chances of cognitive problems in childhood cancer survivors [43–46]. The neuro-cognitive problems could be difficulties in maintaining sustained attention, problems in processing speed, visuo-motor integration, memory and managing executive functions. There may be behavioural changes associated with neuro-cognitive effects and diminished IQ score over time [47]. Some of the cognitive effects start late and follow-up of brain tumour survivors should be holistic so that it covers assessment for physical growth, psychiatric comorbidities, cognitive and adaptive functioning, as well as late medical and endocrine effects. Many cancer centres have specialised late effects clinics for follow-up of these children.

**Cognitive deficits following treatment of brain tumour**

A 5-year-old boy with a 4th ventricle tumour with obstructive hydrocephalus was referred with problems of irritability, frequent tantrums and self-injurious behaviour of hitting himself and also hitting others.

Before the diagnosis of a brain tumour, he used to enjoy watching Cartoon Network on television and used to enjoy playing with his sibling. Following his surgery, the patient had become progressively socially isolated and developed frequent temper tantrums and selfinjurious behaviour. He also became clingy towards his mother that was out of keeping to his usual self.

A generic child psychiatric assessment was completed and the patient’s adaptive functioning was quantified using the Vineland Adaptive Behaviour Scale II using multiple informants. The test results highlighted deficits in several domains in his adaptive functioning. The patient’s parents were encouraged to help him in his day-to-day functioning. They were guided to follow principles of scaffolding to help the patient to relearn his life skills slowly. Parental anxiety was addressed in family therapy sessions. After 1 month, the parents reported that the child’s clinginess and behavioural problems had reduced considerably. The child and family needed support for several months during and following his cancer treatment.

Ideally, neuro-cognitive assessment of paediatric brain tumours should be done at baseline, prior to commencement of treatment, and following treatment completion and every 2 years during follow-up [3]. This is a specialist area that requires expertise to assess and interpret the neuro-psychological test findings of children and young people with brain tumours.

## Learning to manage children with cancer who have special needs

Oncologists have to occasionally deal with medically ill children who may have pre-existing conditions, such as autistic spectrum conditions, attention deficit hyperkinetic disorders and learning disabilities. The nursing staff and other clinicians in oncology hospitals may not be very familiar with handling these children. The main task at hand for paediatric psycho-oncology clinicians in managing children with neuro-developmental conditions is to make it possible for these children to access cancer care in spite of their disability. It may not be possible to do very elaborate assessments on these children as it is usually done in out-patient community based paediatric mental health teams looking after children with neuro-developmental problems who are not medically ill. The children and young people with neuro-developmental disorders should be preferably looked after by the same nursing and medical staff to ensure consistency, which is often immensely reassuring to them. While verbally communicating with these children, one should use precise, short sentences. Children with autistic spectrum conditions find it difficult to adapt to changes and handle uncertainties. While explaining procedures, care should be taken to avoid ambiguous sentences. Clinicians should not promise anything that cannot be delivered as these children are likely to interpret conversations concretely. To give an example, while planning discharge for a child with autistic spectrum condition and cancer, he should not be told that he will be ‘discharged in the afternoon’ as this leaves him with the possibility of defining afternoon as ‘the time just after midday’ and get upset if it gets delayed any longer. Rather, if given a range with a reason, he will likely take it better, even if it has to change slightly. Children with autistic spectrum condition who have developed cancer once sensitively engaged with paediatric oncology services may become easy to handle with every progressing month.

## Helping children to get back to normal life and promoting healthy behaviours and lifestyle following recovery

After months of frequent hospital stays, being away from school with very little push towards achieving educationally, most children and young people need some support to get back to school or college. Difficulties in getting back to school could be due to a plethora of reasons like developing low self-esteem during the course of treatment, body image issues related to hair loss or surgery, sense of impending failure regarding competing with peers in academics and extra-curricular activities, shame related to the prospect of joining a class younger than his peers and parental anxiety in allowing the child to be back in school [48]. Many parents and young people are fearful that the cancer may come back and often get back to their oncologists even for minor health issues that are part of normal growing up [49]. Clinicians need to address these anxieties in a systemic way with the child and his or her family members. Conducting group sessions for children who have completed treatment may help the children in getting back to life and learn from each other. During follow-ups of childhood cancer survivors, monitoring of emotional health is thus as important as the physical parameters [50]. Children should be encouraged to maintain a healthy lifestyle like exercise regularly, have a balanced diet and avoid addictive substances like tobacco. Liaison with school nurses and teachers often helps in integrating children back to peer groups. Long-term follow-up of childhood cancer survivors and addressing their emotional needs as adults has been also commented upon [51].

## Psychological need to have discussions regarding fertility preservation in young people with cancer

The effects of cancer and its treatment on the fertility of children and adolescents when they grow up has been an area of concern for paediatric cancer survivors and their families. The need to address these early on and discuss potential solutions is increasingly becoming important with much better outcomes of childhood cancer in the past two decades. There are now several guidelines that address various aspects of fertility preservation for childhood cancers [52, 53]. Clinicians need to be comfortable in discussing these sensitive issues in an age-appropriate manner, involving the young person whenever suitable and liaise with reproductive medicine specialists. It may be particularly helpful for mental health professionals to be involved in discussions on fertility and fertility preservation for children with cancer as this is often a neglected area of discussion, a pressing concern for many parents and often intertwined with issues related to sexual identity, intimacy and normal sexual exploration [54]. In situations where fertility preservation is not possible, psycho-oncologists need to be ready to engage with the patient and his or her family to address any anticipatory grief related to organ or functional loss.

## Looking after the emotional needs of siblings and parents

Children with cancer need more attention from family members and this may have its own inherent difficulties, particularly in families having more than one child. Parents of children with cancer may be going through significant marital stress, the degree of which may be related to the perceived level of threat due to the cancer and the number of negative events during the course of treatment [55]. Research also indicates that siblings of young patients may require psychological support [56]. Paediatric oncology teams if possible should try to engage the family of a sick child as a unit. They should assess the emotional needs of other children in the same family and be in a position to help all those who are in contact with the child with cancer [57]. They may become fearful that cancer is a contagious disease, feel lonely in a household busy with the child who is sick, feel guilty that his sibling has cancer and develop intense anticipatory grief when the family is facing bereavement. Family members being open about discussing ‘cancer’ can help siblings to get rid of irrational fears. Whenever possible, the adults should make some time for the other children in the family, acknowledge their feelings and tell the siblings that they are equally loved as the child who is sick. Healthy siblings should be encouraged to help other people in the family whenever possible to make them feel part of the family. They should be encouraged to do things they enjoy, take care of themselves, seek help from outside the immediate family if there are neighbours or members of the extended family who are happy to help [58]. When the above ways are not deemed sufficient in dealing with the emotional needs of the sibling, they may need independent professional assessment and management by child mental health professionals. Similarly, some parents will need specialist support and psychological therapy for their own emotional distress. Post-traumatic stress disorder has been reported in parents of children undergoing treatment for childhood cancer [59] and would benefit from specialist mental health inputs.

## Psychological challenges of looking after children who have refractory disease

In spite of the best possible care, some children will have refractory disease in a paediatric oncology setting. This is understandably upsetting for the young people and their families. Refractory disease may not necessarily be immediately terminal. Oncology clinicians often identify psychological distress in children undergoing palliation based on their observation [60]. Psychologically supporting these families is important. Most societies have a taboo about discussing about children’s death and quite rightly would go to any extent to prevent children from dying. However, many paediatric oncologists find themselves in the role of providing paediatric palliative care services for a patient whom they have treated earlier.

At the very end of life, when providing comfort-care is the primary goal of treatment, the emotional impact of impending death can be quite traumatising for everyone. Young people are often able to perceive that their cancer is not responding to treatment and even anticipate imminent ‘end of life’, based on the progression of symptoms and how adults behave with them [61]. For some young people, it is important that palliation is discussed with them so that their concerns are addressed [15]. The two issues that young people are often worried about at this stage are (a) trajectory of physical symptoms such as pain and (b) thoughts on their ‘succession’ around things they hold close to their heart, e.g. who will look after their favourite pet when they are gone. Specialised bereavement focused therapy may be needed for some families to come to terms to with the reality of losing a child. Clinical teams need to be supportive and at the same time be able to give space to the family to grieve, which is often a very private emotion [62].

## Promoting emotional wellbeing in staff looking after children with cancer

One important aspect of paediatric psycho-oncology is to identify staff burnout in teams working with children with cancer [63]. There is a deep-rooted belief that children must not die, and all oncologists do everything possible to save a child. While working with the dying, in end-of-life situations, teams may go through feelings of denial, anger, shame and sadness along with frustrations for having ‘failed’ in some way [64]. Following bereavement, one may be under pressure to move on quickly without getting an opportunity to acknowledge the emotions of loss. These unconscious feelings may accumulate and lead to bigger problems later on. Burnout is usually associated with a gradual loss of satisfaction at work and often accompanied by frequent anger outbursts and also minor health problems like headaches, backaches and stomach aches. The various reasons for professional burnout in paediatric oncology staff as summarised in a review [65] published in 2009 include: (i) observing a child’s pain of death, (ii) caring for a dying child, (iii) having to deal with the event of death, (iv) increased workload, (v) lack of resources, (vi) inadequate support from managers and people who organise the services, (vii) poor working relationship with colleagues, (viii) being involved in delivery of inadequate or inappropriate care. It is important that paediatric psycho-oncology professionals promote psychological wellbeing within the teams to detect burnout early and provide a non-judgemental support for themselves and their colleagues. However ‘stress’ is not the only emotion associated with paediatric oncology. Being a cancer clinician looking after children can be a deeply satisfying and meaningful experience as well [66].

## Models of providing psychological services for children with cancer

The best model for providing paediatric psycho-oncology services is an ‘integrated model’ with paediatric oncologists and mental health professionals working collaboratively. A quasi-liaison model may meet unmet mental health needs of patients in a general hospital better than a ‘on demand consultation-only’ model [67]. It is better to have an in-house department of paediatric liaison psychiatry where child psychiatrists work hand in hand with clinical psychologist for children with cancer. The paediatric psycho-oncology team should be able to learn from on-going reflective practices, provide training and respond to referrals quickly. There should be a process to maintain continuity of care for a particular child who is requiring input from mental health professionals. Specialist paediatric psycho-oncology services should be provided by trained and qualified professionals.

## Conclusion

With improved childhood cancer survival rates, the focus of cancer care has moved beyond the treatment of the biological disease. Training in handling psychological aspects of paediatric cancer will make care more holistic. Learning is a life-long process and most clinicians learn from their own day-to-day practice and from colleagues whom they happen to observe. Having access to additional training opportunities will give the professionals a formal structure to improve their skills. A tiered training programme will likely be more effective when matched with the training needs of a particular individual in a given institution. This will also require training programmes to improve themselves based on feedback from the learners. A paediatric psycho-oncology initiative in a children’s cancer hospital should influence policymakers to create an employee friendly workspace that looks after the emotional wellbeing of employees, including that of doctors, nurses and other staff who tirelessly embark on doing an extremely difficult job with exceptional dedication. Only emotionally healthy staff will be able to deliver emotionally attuned care to young people and their families.

## Conflicts of interest

The authors have no conflicts of interest to declare.

## Funding statement

The authors did not receive any funding support for writing this article.

## Figures and Tables

**Table 1.** Ways to manage therapeutic alliance with children.

Common problems during interviewing	Solutions and ways to tackle communication issues
Lack of private space for interviewing	Children may be sensitive to interacting in public and clinicians should be able to offer them appropriate interview personal space.
Rushing through	Interview should proceed at a gentle pace. If needed, more than one interview may be scheduled.
Being excessively directive	Children and young people do not like to be told what to do. Communication should be such that children feel that everyone is in the same side and fighting the cancer together.
Value judging	It is important not to be judgemental while assessing young people and their families. Clinicians should avoid using labels as ‘a difficult family’ as this may unconsciously affect the quality of the care delivered.
Premature reassurance	Clinicians should avoid reassuring pre-maturely regarding symptom resolution, time of discharge, etc. with young people.
Fostering dependence	Children should be encouraged to think for themselves to help them make decisions about their lives as much as is feasible.

**Table 2.** Referral patterns and psychiatric diagnosis in a children’s cancer centre over 20 months.

Reasons for referral (n = 122)	ALL ^1^ (56/122) 46%	AML ^2^ (11/122) 9%	Lymphoma (15/122) 12%	Brain tumours (19/122) 16%	Sarcomas (6/122) 5%	Others (15/122) 12%
Adjustment disorders (18%)	7	4	3	1	3	4
Behavioural problems (8.2%)	4	2	4	0	0	0
Procedural anxiety (8.2%)	7	1	0	0	0	3
Other anxiety problems (15.6%)	14	0	1	3	0	1
Organic mood disorders (4.9%)	0	0	0	5	0	1
Depression (20.5%)	10	2	6	1	3	3
Neurodevelopmental disorders as ASD^3^ and ADHD^4^ (11.5%)	13	0	1	0	0	0
Neuropsychological testing (7.4%)	0	0	0	9	0	0
Pre-transplant psychological assessment (4.9%)	1	2	0	0	0	3

1 ALL (acute lymphocytic leukaemia);

2 AML (acute myeloid leukaemia);

3 ASD (autistic spectrum disorder)

4 ADHD (attention deficit hyperactivity disorder)
